# Toward enhanced decentralized palliative care services in Neno District, Malawi: a qualitative study.

**DOI:** 10.1186/s12904-024-01455-x

**Published:** 2024-05-23

**Authors:** Atupere S. Phiri, Manuel Mulwafu, Haules Robbins Zaniku, Moses Banda Aron, Judith Kanyema, Stellar Chibvunde, Enoch Ndarama, Grace Momba, Fabien Munyaneza, Lameck Thambo, Chiyembekezo Kachimanga, Beatrice Matanje

**Affiliations:** 1 Partners in Health, Abwenzi Pa Za Umoyo, PO Box 56, Neno, Malawi; 2 Neno District Health Office, Ministry of Health, Neno, Malawi; 3 School of Global and Public Health, Kamuzu University of Health Sciences, Blantyre, Malawi; 4 Research Group Snakebite Envenoming, Bernhard Nocht Institute for Tropical Medicine, Hamburg, Germany; 5 Palliative Care Association of Malawi, Lilongwe, Malawi

**Keywords:** Palliative Care, Health services accessibility, Health personnel, And Rural Health Services

## Abstract

**Background:**

Palliative care remains key in assisting patients who have life-threatening conditions. In most low- and middle-income countries, it is often offered through a centralized system with limitations, including Malawi. In 2014, the World Health Organization called for improving palliative care access through primary health care and community models. Malawi and Neno District subsequently decentralized palliative care delivery to local health centers. This qualitative study explored the decentralization of palliative care services in Neno District, Malawi.

**Methods:**

The descriptive qualitative study was conducted between 2021 and 2022 in two conveniently selected health centers providing palliative care in the Neno District. Fourteen healthcare workers were purposefully selected to participate in two focus groups. Fifteen patients were conveniently selected and participated in three focus groups. Data was analyzed using deductive and inductive approaches. Focused group discussions were conducted in Chichewa (Malawi’s official local language), audio recorded, transcribed, translated into English, and analyzed thematically.

**Results:**

Four main themes emerged from the focus groups. Patients described positive relationships with healthcare workers built on trust and holistic care over time. Accessing care included transport, social support, time constraints, and distance issues. Facilities effectively responded to needs through coordinated care and follow-up. Decentralization was perceived to benefit patients by reducing travel challenges and improving local access to efficient and inclusive palliative care services. However, challenges with resources, distance, and social support remained. Limitations in sampling and missing participant details necessitate further research with broader sampling.

**Conclusion:**

Overall, the study provides empirical evidence that can optimize palliative care delivery in similar low-resource contexts by informing policies to address barriers through decentralized approaches.

**Supplementary Information:**

The online version contains supplementary material available at 10.1186/s12904-024-01455-x.

## Background

Palliative care is an approach that improves the quality of life of patients (adults and children) and their families who face problems associated with life-threatening illness [1–4]. It prevents and relieves suffering by early identification, correct assessment, and symptom control, including pain and other problems, whether physical, psychosocial, or spiritual [5].

Globally, 40% of nations indicated that palliative care was provided (having reached at least 50% of those in need) in a community- or home-based setting. In comparison, 39% reported that it was available in the primary healthcare [6]. Only 5% of the estimated 56.8 million individuals in need of palliative care each year have access to it; most individuals reside in low- and middle-income countries (LMICs) [7–9]. According to estimates from the World Health Organization (WHO), 98% of children in need of palliative care reside in regions where these facilities are non-existent in the majority of LMICs, especially in Africa [10]. Palliative care was more widely offered in primary health care settings (19% of low-income countries), Malawi not spared, than in community- or home-based settings [6].

In Malawi, Palliative care services are provided to a relatively small population facing irreversible, progressive illness during what should be their most economically productive years [11], and only 58% of the target population has been reached in the 94 established palliative care sites in the country available at all levels of care [8, 12]. In the Neno district, the palliative care program registered 921 out of the 1,380 target population at the end of 2021 [1, 13].

In 2014, the first-ever global resolution on palliative care, World Health Assembly (WHA) resolution WHA67.19, called upon the World Health Organization (WHO) and its member states to improve access to palliative care as a core component of health systems, with emphasis on primary health care and community/home-based care model [9]. In the same year, Malawi adopted the resolution and decentralized the responsibility to the health centers as primary implementers of palliative care service delivery, and the Palliative Care Programme of the Neno District Health Office followed suit in 2020 [14].

Using Deming’s Plan, Do, Check, and Act (PDCA) Model cycle of continuous improvement [15], the Neno palliative care program embarked on decentralizing palliative care services to improve the uptake and quality of care provided to patients with life-threatening illnesses. With the approval of the Ministry of Health through the Neno District Health Office, two health centers, Ligowe and Magaleta, were identified as the first sites for decentralization.

Decentralization included four steps: (1) needs assessment to assess capacity building requirements, availability of essential medical supplies, infrastructure capacity to accommodate palliative care patients, and an estimation of potential palliative care patients using patient diagnoses recorded in the facility outpatient registers (OPR), (2) orientation of the healthcare workers in health centers on the principles of palliative care, (3) clinic setup, and (4) monthly mentoring for six months.

All stable ambulatory patients from the Ligowe and Magaleta catchment areas were transferred from the Neno District Hospital static clinic to their nearest facility for follow-up. In 2021, a year after decentralization, it was noted that the program had registered a lower patient uptake (less than 1% of the population required in a given catchment area), as suggested by WHO [14, 16] [14, 16]. . A year later, a study explored the perceived benefits, challenges, and opportunities of decentralizing palliative care to primary-level health facilities.

Therefore, the study explored the effectiveness of palliative care service decentralization by examining the interaction between healthcare workers and patients. It assessed service agility and reliability post-decentralization and examined perceived benefits, challenges, and opportunities. These objectives provided insights into the impact of decentralization on palliative care delivery, focusing on the Neno District in Malawi.

## Methods

### Study design

This descriptive qualitative research employed a deductive and inductive approach to data analysis [17, 18]. It aimed to explore the effectiveness of service decentralization by understanding the interaction between healthcare staff members and patients, ascertaining the agility and reliability of palliative care services, and evaluating the efficiency of palliative care service decentralization.

### Setting

Neno is in the southern region of Malawi, one of the country’s poorest districts. In 2022, the Neno district had an estimated population of 138,291 [19]. Most of the Neno population lives in rural areas and relies on subsistence agriculture as their primary source of income [20]. According to the Malawi National Statistical Office (NSO), the Neno district’s poverty rate was approximately 75% in 2016 [20, 21].

The district has 15 health facilities that provide primary and secondary care to its residents [21]. Neno District Hospital, five health centers (Dambe, Nsambe, Matandani, Neno Parish, and Margareta), and one dispensary (Ligowe) that covers the western side. Lisungwi Community Hospital, three health centers (Luwani, Chifunga, and Matope), and four dispensaries (Midzemba, Zalewa, Nkula, and Tedzani) covering the eastern side. Tedzani is a co-shared facility between the Blantyre District Health Office and the Neno District Health Office regarding administration and management.

Since 2009, the Neno District Health Office has provided palliative care services only in one main referral hospital until 2018, when services were decentralized to the other referral hospital. In 2020, the service was further decentralized to two health centers: Ligowe and Magaleta. These are government-owned public health facilities under the Neno District Health Office. In 2018, the National Statistical Office (NSO) estimated that Ligowe and Magaleta serve a population of 10,941 and 8,723, respectively [22]. Most of the services are government-sponsored, while some are supported by a nongovernmental organization called Partners In Health/Abwenzi Pa Za Umoyo (PIH/APZU) Malawi, palliative care being one of them. Data was collected in a private room or at the nearest health facility. Only the research assistants (RAs) and participants were present during data collection.

### Target population

The target population was all healthcare workers in the Ligowe and Magaleta health centers and all adult patients (18 years and older) who received palliative care services in the Ligowe and Magaleta health centers at the time of data collection. In the study, healthcare workers referred to formally trained and informally trained healthcare providers, including those in the laboratory (on-job trained), ground labour, pharmacy (on-job oriented), clinicians, nurses, and hospital attendants.

### Sample size and sampling technique

Participants were both purposefully and conveniently selected to form five FGD groups. For the healthcare workers at the health centers, two FGDs were conducted, one at each facility. The facility in charge purposefully chose participants based on the staff members’ duty stations. The selection was based on workstations directly in contact with palliative care patients. For patient participants, two male and one female FGDs were conducted, with participants conveniently selected based on who came first to the facility to form a group of 4–6 participants based on the number of patients enrolled.

### Inclusion and exclusion criteria

The inclusion criteria were (a) healthcare workers in the two health centers and (b) all adult palliative care patients who received palliative care services at the Ligowe and Magaleta Health Centre clinics during data collection. The exclusion criteria included (a) patients who were incapacitated at the time of data collection and, (b) patients aged less than 18 years old, (c) All healthcare workers who were not in close contact in their work with palliative care patients.

### Data collection

Patients were contacted face-to-face and were informed about the study by community health workers before they were interviewed. Upon agreement, the patients were told to come to the health center for interviews. The RAs collaborated with the facility in charge, who briefed the healthcare worker participants, scheduled the interview day at the health center, and helped identify the patient participants. The study participants knew the questions during the interview sessions. All invited participants agreed and took part in the study.

Two research assistants (RAs) were hired and trained to administer the FGD guide. The guide was translated from English to Chichewa by a palliative care expert and verified by a qualitative research fellow from Partners In Health/Abwenzi Pa Za Umoyo (PIH/APZU). A pilot FGD was conducted with one group of patients and one group of healthcare workers to test the questions. Two RAs, a qualitative research fellow and a palliative care expert, then reviewed and revised the translated Chichewa question guide based on pilot feedback from the RAs. The translated questions were checked to ensure accurate meaning compared to the English version. Data from the pilot study were not included in the main study.

The RAs did not know the participants before the data collection. Before involving the participants in the study, the information sheet was read aloud and shared with them. Then, the participants were asked to sign or provide a thumbprint to confirm their willingness to participate in the study. No repeat FGDs were needed. The two RAs conducted the interviews and audio-recorded the FGD sessions in the local language. During the interview, one acted as an observer while the other interviewing participants as moderator. On average, the FGD lasted about 90 min. The RAs then transcribed and translated the audio recordings into English.

### Data analysis

Participants’ demographic data was analysed using Microsoft Excel, and counts and percentages were reported. The R.A. transcribed the recorded interviews into Chichewa and translated them into English. The transcribed data was never referred back to participants for corrections or their review. Before developing the codes, AP listened and verified that the recording was transcribed according to the recorded audio. Three researchers (AP, HRZ, and MM) read two transcripts, developed a codebook using phrases, and simultaneously coded sample interviews. Then, the three later converged to resolve any discordant codes. The codes were categorized using the framing and linking approach [17, 18]. To accomplish this, the study utilized Dedoose software (v 9.0.107) for sorting data, and the team iteratively read and analyzed data, continually discussing and refining it until four themes were developed. In the final report, the study adhered to the guidelines provided by the ‘Consolidated Criteria for Reporting Qualitative Research’ (COREQ) [23].

## Results

The study utilized a qualitative research design to explore the experiences and perspectives of key stakeholders involved in the decentralization of palliative care services in Malawi. No participant refused or withdrew from participating in the study. A total of 29 individuals participated across two rural health centers – Ligowe and Magaleta. Specifically, 14 healthcare workers were engaged through focus group discussions, with the majority (*n* = 10, 71%) being male participants from roles such as nursing, pharmacy assistants, laboratory assistants, security, and community health work supervisor. Additionally, 15 palliative care patients at these facilities participated in separate focus groups. Overall patient enrollment across the sites was 18, of which 15 participated in the study with more than half (*n* = 9, 55.6%) were male. While Ligowe Health Center had a slightly higher representation of both staff and enrolled patients, the study drew from a diverse range of viewpoints to understand how decentralization has impacted service delivery from the perspective of both healthcare providers and the recipients of palliative care in these rural Malawian communities (Table 1).

**Table 1:** Demographics of the participants

	Ligowe *N* (male)	Magaleta *N* (male)	*N* (% - male),
Total number of healthcare worker participants	8 (4)	6 (6)	14 (71)
Nurses and clinicians	2 (1)	1 (1)	3 (60)
Pharmacy assistants	1 (1)	1 (1)	2 (100)
Laboratory assistants	1 (0)	1 (1)	2 (50)
Security guards	1 (1)	1 (1)	2 (100)
Community Health Workers	2 (1)	1 (0)	3 (100)
Community Health Worker Site Supervisor	1 (0)	1 (1)	2 (50)
Total number of patients enrolled at each facility	11 (6)	7 (4)	18 (55.6)
Total number of patients that participate in the patient FGDs	11 (5)	4 (4)	15 (60)

The interviews led to developing 610 excerpts and 49 codes and identified four key themes (S2) interconnected with palliative care services. The first theme, “patient and healthcare worker relationship,” focuses on courteousness, privacy and confidentiality, time spent per consultation, and services beyond patients’ expectations. The second theme, “perceived benefits of palliative care program decentralization,” explores advantages such as reduced travel distances, shorter waiting times, and short distances to the clinic. The third theme, “patients’ perceived challenges in accessing palliative care services,” addresses obstacles such as travel costs and the need for increased social support. Lastly, the fourth theme, “facility responsiveness to patients’ needs,” underscores the importance of standardized screening protocols and the provision of equitable care.

### Theme one: patient and healthcare worker relationship

Some participants viewed the service offered near their communities as a good development as they could access it nearby. These patients-provider relationships were seen as a value addition to service accessibility, time allotted for presenting problems, helping patients with courtesy, patient-centered care, privacy, and confidentiality. These patient participants expressed gratitude for how they were treated when seeking palliative care services. This gratitude was evidenced by what one of the patient participants had to say;

*“The healthcare workers at this facility are very friendly and allow me to talk freely in a way that allows me to express my [problems and concerns] without being interrupted.”* (Magaleta Male Patient # 02).

*“[We] can see with the way things [demonstrating how worsted he was] are; that is why they [nurses and clinicians] give [us] the [option] of being two when [we] are [going to] the consultation room. [We] are told that our information will not be disclosed to anyone. Therefore, our privacy is assured as [we] talk inside [closed the doors]. Our privacy is more valued by these doctors here [clinic].”* (Ligowe male patient # 03).

The healthcare worker participants also ensure privacy and confidentiality by consulting patients inside consultation rooms. This practice was evidenced by what one participant had to say;

*“[We] do not see patients in an open space; there is a room where patients consultations are done for [the] sake of privacy. This [pointing at the consultation room] room is where [patients] are seen alone or with their caregivers to discuss the [complaints or condition] the patient is experiencing.”* (Ligowe HC worker # 02).

### Theme two: perceived benefits of palliative care program decentralization

Some participants describe how significant it meant to move the program closer to communities. To patient participants, the move meant reduced transport costs to and from their homes, which helped them cut travel costs. Some participants were unhappy as traveling to Neno District Hospital Clinic meant spending much of their day at the hospital, and some participants enjoyed the fact that they could travel a short distance to the clinic. Some of the patient participants had to say;

*“………having the services right here [at the clinic] is something nice because it was hard for [me] to leave for Chikonde [Neno District Hospital Clinic] for the clinicians to refill my medications. At times, [I] could find that the [severity] of the illness is so intense that [I] cannot manage [do some peace work] to find the money for transport [for my caregiver and myself] to go to [Neno District Hospital Clinic] [for] medications. In such instances, [I] end up [worsening at home] since [money for] transport is unavailable. Therefore, [we] are thankful that the palliative care service has come close to our community.”* (Ligowe male patient # 03).

*“…… when [we] [used to receive] care at Neno DHO, [many patients met] there. [We] would go in the morning and return late in the afternoon, around 01:00 PM and 02:00 PM. Here [referring to the health center], there are few of us patients at [a time], and [we] receive assistance in good time and leave [for homes] in good time.”* (Magaleta Male Patient # 02).

### Theme three: facility response to patients’ needs

Some participants observed that the facilities were more patient-focused, had good rapport development, and were screened thoroughly, including screening for other conditions that were not their main reason for seeking medical care. Participants praised the practice, saying it promoted rapport establishment and standardized care. One of the patient participants had to say this:

*“Once [we] arrive here [clinic], they assist us well. When [we] have arrived, we are weighed on the scale. After being weighed, they [checked] our vital signs, and [we] are asked questions about how [we] feel in our bodies. When this is done, [we] are told to go to the doctor, [who] decides where [we] should go next.”* (Ligowe Male Patient # 03).

### Theme four: challenges in accessing palliative care services

Even though many participants, both patients and healthcare workers, had viewed decentralization to the health center as a good development, some participants still felt that this decentralization was partial. Participants who viewed decentralization partially cited reasons such as distance remaining challenge. Some noted that social services needed to be improved at the health centers. Others complained about essential drugs needing to be stocked consistently, leading to patients traveling long distances without plans if they needed specific medications or social support. Some of the patient participants had to say this:

*‘Where I stay is far, and the means of transport are [complicated] even though the services are now closer to us. When [I] am having body pain, walking a long distance is hard [for me]. [This makes me] walk [slowly to get to the hospital], and [I] rest [often along the way] to gain strength. These are some of the problems [I] still find.”* (Ligowe Female Patient # 03).

*“Let [me] explain [this way] that what [this] woman is saying is true: [we] are receiving drugs, but in terms of food, we do not have the strength to do peace works on our own or farming to bring food to our tables. [For instance, we] did not do farming last rainy season. However, [we] are still receiving drugs and are advised to eat enough before taking medications. On this [emphasizing a point], I am begging the officials at the district level for them to think of us in terms of food.”* (Ligowe Female Patient # 05).

In agreement with patients’ sentiments, healthcare workers viewed the decentralization as incomplete, as some essential services remained at the referral sites. Occasionally, these services could necessitate healthcare workers to refer the patients to the referral facility for care. One healthcare worker had to say,

“*…. Let me start by saying that [at times] [we] have a drug issue. Yes, let us say some patients have severe pain and need morphine, so currently, in Ligowe, [we] do not keep the drug, and it is [the Neno District team does provide [us] with the [drug]. Therefore, [if we meet] such patients, [we] refer them to Neno DHO clinic to access morphine even POSER support [social support].*” (Ligowe HC worker # 02).

## Discussions

This study aimed to explore the effectiveness of palliative care services from a patient and provider perspective following the decentralization of palliative care services from a district hospital to two rural health centers. Decentralization appeared to improve patient-provider relationships. Patients received palliative care closer to home, reducing cost and travel time for patients and caregivers. Furthermore, patients experienced care that was thorough and standardized. However, both healthcare workers and patients were worried about decentralization concerning some aspects of palliative care, as they experienced that the health centers needed more access to social support and essential medication.

The study’s findings illuminate a nuanced exploration of the symbiotic relationship between patients and healthcare workers within palliative care services. Research by Street et al. (2014) underscores the pivotal role of a positive patient-provider relationship, emphasizing its influence on service accessibility and patient satisfaction [24]. The study aligns with the sentiments expressed by participants, where courteous treatment, patient-centered care, and respect for privacy contribute to robust healthcare accessibility. As highlighted by participants, the proximity of services to communities resonates with findings from a study by Saurman (2016), which emphasizes the importance of geographical accessibility in healthcare utilization [25]. Patient testimonials provide experiential evidence supporting the positive impact of friendly and open communication on patient well-being, aligning with research by Beach et al. (2006) [26]. Furthermore, the commitment of healthcare worker participants to ensuring privacy and confidentiality within consultation rooms aligns with the principles of patient-centered care advocated by Varkey (2021) [27]. The discussion extends beyond individual experiences, drawing on established literature to enrich our understanding of the dynamics shaping palliative care experiences and emphasizing the broader implications for healthcare practices to service accessibility.

The study’s findings further demonstrate the benefits of palliative care program decentralization aligning with existing literature. In a publication by Robert et al. (2021), the positive impact of bringing health services closer to communities was highlighted, resonating with our participants’ experiences [28]. The observed reduction in transport costs, echoing the study by Shrank et al. (2021), underscores the financial burden associated with distant healthcare access [29]. These findings contribute to the broader literature, offering insights for program planners and stakeholders seeking to enhance palliative care accessibility and effectiveness in community-based settings.

The theme “facility response to patients’ needs,” as identified in the study, is substantiated by existing research on patient-centered care. Notably, studies like Ford (2004) underscore the positive outcomes of robust physician-patient relationships, aligning with the observed development of good rapport in the facilities [30]. The emphasis on interpersonal connections reflects the importance of patient-centered practices in healthcare. Moreover, the comprehensive screenings implemented in the facilities, extending beyond the primary reason for seeking care, align with recommendations from authoritative bodies such as the Institute of Medicine (IOM) [31]. The IOM advocates for patient-centered, evidence-based healthcare, resonating with global initiatives by the World Health Organization (WHO) and the Agency for Healthcare Research and Quality (AHRQ) to promote equitable healthcare and reduce health disparities [32, 33].

Challenges in accessing palliative care services reveal concerns despite the optimistic view of decentralization. Participants cited distance as a persistent challenge, supported by Bossert et al.‘s (2003) acknowledgment of distance as a barrier even with decentralization [34, 35]. The social support participants mentioned align with Andermann (2016), emphasizing the need to address social determinants for effective healthcare [36]. The inconsistency in essential drug stocking at health centers echoes supply chain challenges noted by Vledder (2019), highlighting the practical issues in healthcare supply chains [37]. These references provide a comprehensive understanding of the challenges in decentralized health services, including in rural settings.

A previous study on service decentralization has emphasized the importance of building a robust social support system to achieve positive outcomes [38]. The study also found that patients in peripheral health facilities face challenges accessing social support compared to those in referral facilities. The lack of a vibrant social support system in rural areas means that palliative care services are not holistic, as psychosocial support services are often unavailable [2, 39, 40]. Therefore, it is imperative for district-level programs to devolve power to rural facilities, allowing them to function optimally and provide comprehensive care that includes psycho-social support services [41–43].

### Strengths and limitations of the present study

Some strengths and limitations of this study were subjected to include;

### Strengths

The study used FGDs to get in-depth views from patients and healthcare workers. Participants were purposefully selected from key workstations to bring diverse perspectives of males and females. The study used a rigorous data analysis process involving coding by multiple researchers and the use of software to ensure reliability. The study also used a reporting method to adhere to international reporting standards of research findings (COREQ guidelines) to improve transparency.

### Limitations

The health centers involved limit the generalizability of the results to other areas. Again, convenience sampling of some patients may have introduced information bias. The study should have collected demographic data reports of participants, limiting understanding of their backgrounds.

## Conclusion

The study explored the decentralization of palliative care in two Malawian health centers through focus group discussions with healthcare workers and patients. Findings provided support for decentralizing services, with improved relationships and accessibility reported. However, resource constraints, distance, and lack of social support highlighted ongoing challenges. While offering insights, limitations in sampling and lack of participant demographics necessitate further research with broader sampling to strengthen understanding of decentralization’s effects. The study contributes empirical evidence to optimize palliative care delivery in similar low-resource settings by informing policies and programs addressing barriers to comprehensive care through decentralized models.

### Electronic supplementary material

Below is the link to the electronic supplementary material.

Supplementary Material 1

Supplementary Material 2

## Data Availability

The datasets supporting the article’s conclusions are included, with hyperlinks to the data where applicable within the references. The datasets used for simulations in the current study are available from the corresponding author upon reasonable request.

## Abbreviations

FGD: Focused Group Discussion
LMIC: Low-Middle Income Countries
WHO: World Health Organisation
WHA: World Health Assembly
PDCA: Plan, Do, Check, Act
OPR: Outpatient Register
NSO: National Statistical Office
PIH/APZU: Partners In Health/Abwenzi Pa Za Umoyo
AP: Atupere Phiri
HRZ: Haules Robbins Zaniku
MM: Manuel Mulwafu
COREQ: Consolidated Criteria for Reporting Qualitative Research
HCW: HealthCare Worker
CHW: Community Health Worker
DHO: District Health Office
COVID 19: Coronavirus Disease 2019
