# Supplementary material for: Toward enhanced decentralized palliative care services in Neno District, Malawi: a qualitative study
Source: BMC Palliat Care. 2024 May 23;23:132. doi: 10.1186/s12904-024-01455-x (PMC11112853; doi:10.1186/s12904-024-01455-x)
Supplement: Supplementary file 1 — Supplementary Material 1 [file 12904_2024_1455_MOESM1_ESM.doc]

## **Focus Group Question Guide for patients and healthcare workers**

1. **General questions for palliative care patients.**

Dear participant,

I thank you for agreeing to participate in the study; this study seeks to evaluate the quality of care following the decentralization of services to the health centers in the Neno District. You are encouraged to ask questions if you need further clarification and understanding regarding the interview and be assured that the information shared will not be disclosed to anybody other than those involved in the research project.

Section 1: Demographic Data.

| **Participants details** | **P1** | **P2** | **P3** | **P4** | **P5** | **P6** | **P7** |
| --- | --- | --- | --- | --- | --- | --- | --- |
| Age |  |  |  |  |  |  |  |
| Gender |  |  |  |  |  |  |  |
| Education Level |  |  |  |  |  |  |  |
| Duration in the program (years) |  |  |  |  |  |  |  |

**Section 2: General questions**

**How is the interaction between patient and healthcare worker at this facility?**

**Guiding questions**

1. How do they handle when they come for medical help at this health center?
   - Friendly toward patients and caregivers
   - Listening to patient’s concerns
2. On average, do you think this HC staff member gives you enough time to talk about your problems or worries?
   - Time spent with one patient.
   - Health workers responding to questions.
3. How do healthcare workers ensure that they provide safety when helping you?
   - Confidentiality and respect.
   - Consultation rooms
4. If one of your friends or family member was a palliative care patient, would they recommend they come to this facility for medical help?
   - Health worker attitude
5. What are some of the reasons patients would opt for herbs and witchdoctors when palliative care services are now at this health center?
   - Cultural beliefs
   - Information sharing to the community served by the health center.
   - Advocacy
     1. **Agility and reliability of palliative care services at this health center.**

**Guiding questions**

- - - - In what ways has the establishment of palliative care services at this facility helped you?
        - Drug supplies.
        - Frequency of visits to facilities.
      - How do you think the health center would be supported by the Neno DHO office to provide good services?
      - Drug supplies
      - In your view, how could the program respond to the social support of vulnerable palliative care patients?
      - How has COVID-19 affected palliative care services to you as patients?
        - Patient attendance at the clinic.
    1. **Effectiveness of palliative care program decentralization.**

**Guiding questions**

1. What could be the barriers facing the community utilizing palliative care services at this health center?
   - - - Community barriers
       - Facility barriers
       - Individual barriers
2. How could the Neno DHO palliative care team support this health center in terms of service provision?
   - - - Patient reviews at the clinic and homebound
3. What do you like most about palliative care service decentralization?
   - Time spent visiting the facility
   - Availability of medical support
4. What do you like least about palliative care service decentralization?
   - - - - Drug supplies
         - Health worker attitude
5. **General questions for healthcare workers.**

Dear participant,

I thank you for agreeing to participate in the study; this study seeks to evaluate the quality of care following the decentralization of services to the health centers in the Neno District. You are encouraged to ask questions if you need further clarification and understanding regarding the interview and be assured that the information shared will not be disclosed to anybody other than those involved in the research project.

Section 1: Demographic Data.

| **Participants details** | **P1** | **P2** | **P3** | **P4** | **P5** | **P6** | **P7** |
| --- | --- | --- | --- | --- | --- | --- | --- |
| Age |  |  |  |  |  |  |  |
| Gender |  |  |  |  |  |  |  |
| Education Level |  |  |  |  |  |  |  |
| Position at Health Facility |  |  |  |  |  |  |  |
| Work experience (years) |  |  |  |  |  |  |  |

**Section 2: General questions**

1. **Interaction between patient and healthcare provider.**

Guiding questions

1. How do you handle patients when they come for palliative care consultations at this health center?
   - Attitude
   - Friendly toward patients and caregivers
   - Listening to patient’s concerns
2. How do you make sure that patients and their relations do understand what to tell them?
   - Health workers spend more time with one patient.
   - Patients are given time to ask questions regarding their conditions.
   - Time spent with one patient
     1. Why that long?
3. How do you make sure that patients are safe?
   - Use of national palliative care guidelines
   - Consultation rooms
4. If one of your friends or family member was a palliative care patient, would they recommend they come to this facility for medical help?
   - Availability of drugs and essential supplies in line with palliative care services.
   - Advocacy
5. What are some of the reasons patients would opt for herbs and witchdoctors when palliative care services are now at this health center?
   - Cultural beliefs
   - Information sharing to the community served by the health center.
   - Accessibility
6. **Agility and reliability of palliative care services at this health center.**

Guiding questions

- - - 1. What impact has the coming of palliative care had on solving some of the problems patients are facing?
         - Drug supplies to patients.
         - Frequency of visits to facilities.
      2. How do you think would the Neno DHO office support the level of care at this facility?
         - Drug supplies
         - Mentorship and supervision
         - Data reviews
         - Patient reviews at the clinic and homebound
      3. How have other programs affected service provisions at this facility?
         - Integration with other services, i.e., NCD/ART program
      4. In your view, how could the program respond to the social support of vulnerable palliative care patients?
         - Food assistants.
         - Renovation of houses.
         - School support to school attending siblings of the patients.
      5. How has COVID-19 affected palliative care service provision to patients in your community?
    - Patient attendance at the clinic.

1. **Effectiveness of palliative care program decentralization.**

Guiding questions

1. How is palliative care service decentralization achieving the intended goal of the program?
   - - - Increase uptake of patients into the program.
       - Retention to care of patients.
2. In the process of implanting decentralization, what are the challenges you face, if any?
   - - - Community barriers
       - Facility barriers
       - Individual barriers
