# Supplementary material for: Toward enhanced decentralized palliative care services in Neno District, Malawi: a qualitative study
Source: BMC Palliat Care. 2024 May 23;23:132. doi: 10.1186/s12904-024-01455-x (PMC11112853; doi:10.1186/s12904-024-01455-x)
Supplement: Supplementary file 2 — Supplementary Material 2 [file 12904_2024_1455_MOESM2_ESM.doc]

## **Codebook**

General Codes

| **Code** | **Description** |
| --- | --- |
| Courtesy of | Participants describe the way healthcare workers welcome patients with dignity. |
| Patient-centered | Participants describe the approach in which care provision is focused on the patient as a whole. |
| Enough time/space | Participants describe the freedom patients have to explain what is troubling them. |
| Privacy and confidentiality | Participants describe the safety of the environment and the information they share with healthcare workers. |
| Rapport | Participants describe the harmonious relationship between healthcare workers and patients. |
| Patients feedback mechanism | participants described the process of having a platform where they voiced their personal experiences with medical personnel at the facility. |
| Trust | Participants describe the confidence they have in healthcare workers at the facility. |
| Holistic care | Participants describe the totality of care they get from the healthcare workers. |
| Follow-ups of patients | Participants describe the monitoring of patients’ conditions after they receive care at the facility. |
| Grateful | Participants appreciate the services they get from the facility. |
| Barriers to accessing palliative care services | Participants describe factors that prevent patients from accessing palliative care services at health facilities. |
| Poor knowledge of palliative care services available | Participants describe the patient's little understanding of palliative care services offered |
| Lack of transport | Participants describe costs incurred in the process of seeking palliative services. |
| Time | participants describe the time spent from arrival at the hospital to the time they were assisted |
| Medical supplies availability | Participants describe the availability of medical supplies for palliative care following decentralization. |
| Long distance | Participants describe how far it is to get to the facility and how hard it is to get to the facility. |
| Follow-ups of patients | Participants describe the monitoring of patients’ conditions after they receive care at the facility. |
| Lack of collaboration between departments | Participants describe the experiences resulting from segmented/uncoordinated services between departments/sections. |
| Following screening protocol | participants describe the step-by-step processes they undergo when they arrive at the facility |
| Provision of social support | Participants describe the support given to the patient for them to be self-reliant. |
| Fear of image tarnishing | participants describe the fear of being labeled negatively in the community |
| COVID 19 misconceptions | Participants describe the myths about COVID-19. |
| Service interruption due to COVID-19 | Participants describe the interruption of services as a result of the COVID-19 outbreak. |
| Time | participants describe the time spent from arrival at the hospital to the time they were assisted |
| Follow-ups of patients | Participants describe the monitoring of patients’ conditions after they receive care at the facility. |
|  |  |
|  |  |
| **Themes** |  |
| **Themes** | **Description** |
| Patient healthcare worker relationship | Participants describe the rapport and trust developed over time with the system. The holistic care and the courtesy call rendered to them whenever they seek assistance at the facility, be it social, psychological, spiritual, or physical. |
| Perceived challenges in accessing palliative care services | Participants described concerns related to transport, provision of social support, time constraints, and long distances. These identified challenges provide valuable insights into the factors that may affect palliative care services being accessible at health centers. |
| Facility response to patients’ needs | Participants articulate the sequential procedures, additional non-medical support, timeliness, and well-coordinated services with established follow-up mechanisms. The focus is patient-centered care, fostering rapport, adhering to screening protocols, and incorporating a patient feedback mechanism. |
| Perceived benefits of palliative care program decentralization | Participants describe decentralization positively, seeing it as a solution to time-related lack of transport, reducing the extensive travel challenges, and appreciating the efficiency of accessing care locally, making the service efficient, accessible, and inclusive in palliative care delivery |
